# Evaluation of pyrolysis chars derived from marine macroalgae silage as soil amendments

**DOI:** 10.1111/gcbb.12722

**Published:** 2020-07-24

**Authors:** Jessica M. M. Adams, Lesley B. Turner, Trisha A. Toop, Marie E. Kirby, Christine Rolin, Emma Judd, Rhiannon Inkster, Lesley McEvoy, Waseem M. Mirza, Michael K. Theodorou, Joseph Gallagher

**Affiliations:** ^1^ Biorefining Group Institute of Biological, Environmental and Rural Sciences Aberystwyth University Aberystwyth UK; ^2^ Agricultural Centre for Sustainable Energy Systems Department of Agriculture and the Environment Harper Adams University Newport UK; ^3^ NAFC Marine Centre (UHI) Scalloway UK

**Keywords:** agronomic performance, biochar, biorefining, fertilizer, seaweed, thermo‐catalytic reforming

## Abstract

Pyrolysis char residues from ensiled macroalgae were examined to determine their potential as growth promoters on germinating and transplanted seedlings. Macroalgae was harvested in May, July and August from beach collections, containing predominantly *Laminaria digitata* and *Laminaria hyperborea*; naturally seeded mussel lines dominated by *Saccharina latissima*; and lines seeded with cultivated *L. digitata*. Material was ensiled, pressed to pellets and underwent pyrolysis using a thermo‐catalytic reforming (TCR) process, with and without additional steam. The chars generated were then assessed through proximate and ultimate analysis. Seasonal changes had the prevalent impact on char composition, though using mixed beach‐harvested material gave a greater variability in elements than when using the offshore collections. Applying the char at 5% (v/v)/2% (w/w) into germination or seedling soils was universally negative for the plants, inhibiting or delaying all parameters assessed with no clear advantage in harvesting date, species or TCR processing methodology. In germinating lettuce seeds, soil containing the pyrolysis chars caused a longer germination time, poorer germination, fewer true leaves to be produced, a lower average plant health score and a lower final biomass yield. For transplanted ryegrass seedlings, there were lower plant survival rates, with surviving plants producing fewer leaves and tillers, lower biomass yields when cut and less regrowth after cutting. As water from the char‐contained plant pots inhibited the lettuce char control, one further observation was that run‐off water from the pyrolysis char released compounds which detrimentally affected cultivated plant growth. This study clearly shows that pyrolysed macroalgae char does not fit the standard assumption that chars can be used as soil amendments at 2% (w/w) addition levels. As the bioeconomy expands in the future, the end use of residues and wastes from bioprocessing will become a genuine global issue, requiring consideration and demonstration rather than hypothesized use.

## Graphical abstract

Lettuce seedlings 21 days after sowing with and without the addition of ensiled, thermo‐catalytic reforming macroalgae char and inorganic fertiliser to the growing medium. Orange square = SoilFixer control char.

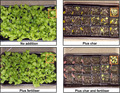

## 1 INTRODUCTION

Macroalgae (seaweeds) have a high productivity rate and do not compete with land demands for food production, fertilizers or fresh water (Adams, Bleathman, Thomas, & Gallagher, 2017; Loureiro, Gachon, & Rebours, 2015; Suutari et al., 2015). As a source of biomass, therefore, macroalgae are a potential feedstock for any number of products without adversely affecting current food production or land use. A key end‐product in demand is that of liquid hydrocarbon fuels. These can be produced by pyrolysis, hydrothermal liquefaction or gasification followed by Fischer–Tropsch synthesis to the liquid fuels (Douvartzides, Charisiou, Papageridis, & Goula, 2019). Thermo‐catalytic reforming (TCR) is a novel process capable of pyrolysing feedstocks with high ash content (Kirby, Hornung, Ouadi, & Theodorou, 2017) such as macroalgae. The TCR process operates through two stages of intermediate pyrolysis (350–450°C) followed by a catalytic reforming step (~700°C). Within this second step steam can also be injected to help volatile gas molecules reform (Kirby et al., 2017) before condensing to generate bio‐oil and syngas in varying quantities with char as a by‐product (Bridgwater, 2012). These fractions have highly variable properties depending on the feedstock used and the processing parameters applied.

The use of char as an agricultural soil amendment has received much attention over recent years. Addition of char to soil is widely reported to provide numerous benefits such as improvements in crop yield, increases in rhizosphere microbial diversity, enhancement of the soil carbon pool and mitigation of greenhouse gas emissions (Jeffery, Verheijen, van der Velde, & Bastos, 2011; Kammann et al., 2017; Kavitha et al., 2018; Kolton, Graber, Tsehansky, Elad, & Cytryn, 2017). However, published work does show some variability in the extent of positive benefits depending on the source of the char, method of production and conditions tested (Jeffery et al., 2011, 2017), with plant responses sometimes less predictable than those using non‐biological amendments such as chemically derived fertilizers (Abbott et al., 2018). Often greater success is achieved using biochars matched to specific crops and the environment they are grown in. The meta‐analysis of literature by Jeffery et al. (2011) identified soil improvements of liming acidic soils and improving water holding capacity as two key biochar benefits, depending on the biochar used and application rate, in addition to increased nutrient availability. They demonstrated that soil types, environmental factors and land management techniques all play a role regarding the effect of biochar on the plant yields, with most biochars giving a positive effect on crop yield and only an undisclosed ‘biosolids’‐derived biochar having a significantly negative one.

Macroalgae differ compositionally from terrestrial biomass when fresh, containing compounds such as laminarin and alginate in browns, carrageenan or agar in reds and ulvan in greens. (Percival, 1979). They also have a higher moisture content than most terrestrial plants, with monthly collections for 2 years of *Laminaria digitata* showing 84%–90% moisture in whole algae (Black, 1950). Similarly, low pyrolysis char (450°C) from a range of red and brown seaweeds collectively differed from lignocellulosic‐derived biochar produced under similar conditions with a greater yield of char, lower carbon content but higher elemental content relative to lignocellulosic‐derived biochar (Roberts, Paul, Dworjanyn, Bird, & de Nys, 2015). Macroalgae chars have a pH between 7 and 11 (Roberts et al., 2015) and are potentially unique materials for soil amelioration, differing significantly from lignocellulosic chars (Roberts et al., 2015; Yu et al., 2017). However, there have been few assessments of the possible horticultural applications of marine macroalgae chars (Bird, Wurster, Silva, Paul, & de Nys, 2012; Roberts & de Nys, 2016; Roberts et al., 2015) and compositional data are limited. Chars derived from cultivated brown and red algae varied depending on the origin of the macroalgae, but could be characterized as having a low carbon content, low surface area and a high N, P, K content, particularly for K.

A limiting factor in using macroalgae as a feedstock is its seasonality. Despite rapid growth rates and high densities within for example, kelp beds (Adams, Gallagher, & Donnison, 2009) at peak times of the year, in the winter months, algae is lost through storms and strong tidal movements which also prevent off‐shore harvesting. Ensiling is a long‐established technique for anaerobically preserving seasonal biomass, primarily as forage for livestock (Keady, Hanrahan, Marley, & Scollan, 2013) but more recently also as a storage preparation prior to bioconversion into products such as biomethane (Janke et al., 2019; Mangold, Lewandowski, Hartung, & Kiesel, 2019; Prade, Svensson, Horndahl, & Kreuger, 2019). Work relating to the ensiling of macroalgae is less common, though it was initially scientifically studied as an ensiling feedstock in the 1950s (Black, 1955). Most academic studies using ensiled macroalgae have been focussed on its use as a forage source, especially for marine molluscs (Ancca et al., 2018; Mardones, Cordero, Augsburger, & De los Rios‐Escalante, 2015; Uchida, Numaguchi, & Murata, 2004) and ruminants (Cabrita, Maia, Sousa‐Pinto, & Fonseca, 2017; Maia, Fonseca, Oliveira, Mendonca, & Cabrita, 2016). Within the bioconversion arena, research has focussed on the effect that ensiling has on methane yields (Herrmann et al., 2015; Milledge & Harvey, 2016), on higher heating values (HHV) and on ash content (Redden, Milledge, Greenwell, Dyer, & Harvey, 2017). In this last study, results showed that though the energy content of the macroalgae changes during the ensiling process, primarily through the loss of mass by leachate, it retained the elemental content, leading to similar ash values for fresh and ensiled macroalgae (Redden et al., 2017). This research complements a review by Milledge and Harvey (2016) which identified ensiling and gasification as a viable combination which provided a storable biomass feedstock for year‐round use and served as a method for utilizing the whole biomass.

In this paper, we advance the theoretical concepts in Milledge and Harvey (2016) to applied trials and to our knowledge this represents the first reporting of pyrolysis char from ensiled kelp macroalgae; and one of the first pyrolysed macroalgae char plant trials. In this paper, macroalgae harvested at different months within the growing season consisted primarily of identified, different kelp species which were ensiled to enable long‐term storage of the material. This preserved material was then pelleted and pyrolysed using the TCR process with and without steam addition. The residual chars were analysed for proximate and ultimate analyses, then used in plant cultivation trials to ascertain whether the char inclusion benefitted plant germination and growth. Germination and early growth of lettuce seedlings were examined in detail under controlled conditions with and without the different chars and a standard fertilizer. A longer‐term growth trial subsequent to germination was also conducted, with trays (mini‐swards) of transplanted annual ryegrass, *Lolium temulentum* L. Findings thus report, for the first time, on both the algal silage char composition and its effect on initial and subsequent growth of two nutrient‐demanding crops.

## 2 MATERIALS AND METHODS

### 2.1 Macroalgae collection, algal ensiling and preparation of pellets for pyrolysis

Eight macroalgae collections were made from the shore (beach‐harvests) or a boat (rope or longline harvests) within western Shetland (United Kingdom) in May, July and August 2015, with each harvest period covering up to 3 days. These were sourced close to the NAFC Marine Centre as follows: (a) Mixed kelp biomass from a beach‐harvest following the receding tide (predominantly *L. digitata* and *L. hyperborea*; Trondra, 60°07.370′N, 001°16.320′W; May, July, August). (b) Material naturally settled and growing on mussel production ropes (predominantly *S. latissima*; Lea of Trondra, 60°07.119′N, 001°16.456′W; May, July, August). (c) Material harvested from preseeded (cultivated) macroalgae longlines (*L. digitata*; Sandsound South, 60°13.384′N, 001°22.267′W; July and August only; no May silages were prepared from this location as there was insufficient biomass on the lines to harvest). For each site and on each occasion, approximately 200 kg of algal biomass was harvested and returned within 3 hr to the NAFC Marine Centre. Initial processing involved spreading the algal biomass on plastic sheeting covering a concrete floor in an enclosed building held at ambient temperature overnight (12 hr) prior to further processing. Harvested material was chopped using a Viking GE‐250 garden shredder (Viking, GmbH) which reduced the average frond size to discrete pieces of approximately 3 × 2 cm (length × width). Once chopped, the silage additive, Safesil (Kelvin Cave Ltd.) was used to assist in the preparation of silages. It was applied with a watering can at the manufacturer's recommended dose rate of 4 L/t fresh weight to quantities of chopped algal biomass churning in a 136 L capacity Belle Minimix 150 electric concrete mixer (Belle Engineering Ltd). The treatment time for each load of the cement mixer was approximately 10 min. Sufficient algal biomass was treated from each collection to fill two replicate 225 L conical bottom round Paxton tanks (Stanford Products Ltd); each tank contained 50 kg of treated algal biomass. Tanks were lined with a 914 × 1219 mm heavy duty polythene bag (Kite Packaging) which was compressed to evacuate air, sealed with black PVC silage tape 100 × 33 mm (Sticky Products Markham) and covered in 10 kg of dry sand. A total of 16 silos were prepared; these silos were opened 90 days later and the ensiled macroalgae was sampled for pH as detailed below. The remainder was dried to a constant weight at 60°C (4 days), milled using a SM100 knife mill (Retsch) and pelleted with 2% (w/w) vegetable oil to prevent sticking in the mill using a Hi Flow Simon Baron pellet mill (Equipment supply services) through a 6 mm die to >30 mm length at IBERS, Aberystwyth. Pellets generated were of low tensile strength.

### 2.2 Silage pH analysis

All silages produced small quantities of leachate which were drained upon opening. When opened, one of the May mussel rope silos was covered in mould; this silo was discarded and not subsampled and all other containers were analysed for pH as below. Each silage pH was recorded by removing 15 samples, consisting of three samples taken from five layers equally spaced throughout the biomass. At each subsample point, 10 g of ensiled material was removed and placed into a Stomacher bag with 90 ml of deionized water. The sample was pummelled using a Seward Stomacher 400 Circulator (Seward) for 3 min at 230 rpm. The pH was determined using a calibrated combination pH probe (Jenway 3505 pH Meter; Jenway).

### 2.3 Char production

Pellets prepared as detailed above were sent to the Fraunhofer Institute for Environmental, Safety and Energy Technology, UMSICHT, Institute Branch Sulzbach‐Rosenberg, Germany for pyrolysis processing to generate char for further analysis. Chars were produced using the Fraunhofer TCR laboratory‐scale plant, a new process that combines an intermediate pyrolysis step with a postcatalytic treatment (reforming). Equipment design including diagrams is available within Kirby et al. (2017). Briefly, the feedstock was fed under nitrogen through the reactor via a screw mechanism operating at 2 kg/hr, providing a residence time of 5–10 min at 450°C before entering a 700°C reformer chamber (residence time 5–10 min). Char was collected from the reformer around low‐ash birch wood chips to absorb the macroalgae char in dust form which would otherwise be lost whilst minimizing result distortion. Two cooling units were used to condense and collect gases, these will be reported in a subsequent publication. In a replicate process, a constant steam flow of approximately 10% (w/w) of the feedstock was injected into the reactor during the process, termed ‘steam reforming’, which increased the relative proportion of gas production, reducing the proportion of char produced. Subsequent reference to material from the TCR process in this article is referred to as ‘regular char’; that which included steam within the reforming stage is referred to as ‘steamed char’.

### 2.4 Silage and char analyses

#### 2.4.1 Proximate and ultimate analysis

Predried pellets and char produced by TCR with and without steam reforming were analysed by UMSICHT according to DIN EN ISO 16948 and DIN 51900 standards to determine the following: elements C, H, N, S, O (by difference); HHV; lower heating values (LHV); water content and ash content. Pellet bulk density calculations were conducted with UMISCHT by measuring the total volume and weight of the pelleted silage samples provided (mean weight 5.8 kg, standard deviation 1.6, values given to 1 decimal place only). Regular char and steamed char bulk density calculations were conducted by IBERS, Aberystwyth University, by measuring the weights of 50 ml untapped char volumes (char particles 0–2 mm diameter).

#### 2.4.2 pH and conductivity of the chars

Methodology was based on work by Li et al. (2013) and Singh, Dolk, Shen, and Camps‐Arbestain (2017). Each biochar sample was milled and sieved to <2 mm. The pH and electrical conductivity (EC) were measured by weighing 2 g of biochar into a container with deionized water at a ratio of 1:5. Samples were shaken (HS 501 digital, IKA) for 1 hr at 250 rpm, prior to centrifugation (Hettich Rotina 46R) at 4°C, 3,490 *g* for 4 min to concentrate the biochar pellet. The pH was measured using an integrated pH and EC Jenway 3540 meter (Jenway), calibrated to pH values of 7 and 10. EC was then measured with the EC probe. All samples were repeated in triplicate.

#### 2.4.3 Elemental analyses

Ensiled, regular char and steamed char material were analysed at the accredited laboratories at IBERS to determine the elemental concentrations of aluminium, barium, cadmium, calcium, chromium, copper, iron, lead, magnesium, manganese, molybdenum, nickel, potassium, sodium, strontium and zinc. A commercially available product, milled SoilFixer Biochar (particles 0–2 mm diameter), produced from UK‐grown coppiced woods using a ring kiln at 400–600°C for 8–12 hr, was also analysed for comparison (SoilFixer Ltd).

For each sample, 1 g material was weighed into 100 ml Kjeldahl tubes and about 15 ml aqua regia (780 ml HCl; 500 ml HNO_3_; 720 ml H_2_O) was added and allowed to soak overnight. Samples were digested on a heating block at 120°C for 3 hr, allowed to cool and then quantitatively transferred to 50 ml volumetric flasks. The solutions were filtered through Whatman No 1 filter paper and then analysed using a Varian Liberty ICP‐AES (Agilent Technologies). Total phosphorus was determined in duplicate according to (Taussky & Shorr, 1953); briefly 150–200 mg char were precisely weighed into calibrated Volac tubes with 1 ml 2M H_2_SO_4_ for 4 hr at 150°C. Tubes were removed, two drops of 30% H_2_O_2_ was added and returned to incubator for a further 2 hr before cooling, making up to 4 ml with deionized water. Quantification was conducted in flat‐bottomed 96 well plates in triplicate with known phosphorus standard series for calibration. Each well contained 50 μl of each of the following: liquid sample, 3% TCA and molybdate solution prepared as Taussky and Shorr (1953). Once added, tubes were left to stand for 15 min at room temperature, to which 50 μl deionized water was added, then read at 740 nm.

### 2.5 Plant trial materials and growth conditions

The chars used in these studies are detailed in Table 1. All chars were milled to give particles of 0–2 mm diameter prior to use in the growth trials to replicate that in the SoilFixer control.

**TABLE 1:** Char materials used in plant growth trials

Char identifier	Source material	Material type	Within lettuce growth trial	Within ryegrass growth trial
RC#1	Beach‐harvest—May	TCR regular char	Yes	*
RC#2	Mussel line collection—May	TCR regular char	Yes	*
RC#3	Beach‐harvest—July	TCR regular char	Yes	Yes, combined with RC#6*
RC#4	Mussel line collection—July	TCR regular char	Yes	Yes, combined with RC#7*
RC#5	Algal line collection—July	TCR regular char	Yes	Yes, combined with RC#8*
RC#6	Beach‐harvest—August	TCR regular char	Yes	See above
RC#7	Mussel line collection—August	TCR regular char	Yes	See above
RC#8	Algal line collection—August	TCR regular char	Yes	See above
SC #1	Beach‐harvest—May	Steamed TCR char	Yes	*
SC #2	Mussel line collection—May	Steamed TCR char	Yes	*
SC #3	Beach‐harvest—July	Steamed TCR char	Yes	Yes, combined with SC#6*
SC #4	Mussel line collection—July	Steamed TCR char	Yes	Yes, combined with SC#7*
SC #5	Algal line collection—July	Steamed TCR char	Yes	Yes, combined with SC#8*
SC #6	Beach‐harvest—August	Steamed TCR char	Yes	See above
SC #7	Mussel line collection—August	Steamed TCR char	Yes	See above
SC #8	Algal line collection—August	Steamed TCR char	Yes	See above
Control	SoilFixer char	commercial wood char	Yes	Yes
No additive	Not applicable	Not applicable	Yes	Yes

Abbreviations: RC, regular char; SC, steamed char; TCR, thermo‐catalytic reforming.

* Material combined in ryegrass trial due to insufficient material available individually. May collections excluded as insufficient material available even when combined.

#### 2.5.1 Lettuce

Green salad bowl lettuce (Mr Fothergill's) was grown from seed in 7 × 7 × 8 cm square black pots. The basal growing medium was 2:1:1 of John Innes seed compost (Westland), grit sand (Kelkay) and medium perlite (LBS Horticulture) respectively; amended with 1.8 g/L Vitax Q4 powdered fertilizer (LBS Horticulture) and/or 5% char by volume. Sixteen seeds per pot were surface sown in a 4 × 4 grid; the pots were watered from below. All pots were placed in a Fitotron‐controlled environment cabinet (Sanyo Gallenkamp; now Weiss Technik UK) with a 16 hr day and white LED lights (280 μmol m^−2^ s^−1^ irradiance at pot height). Trays were rearranged daily to allow for any cabinet variation. Germination was carried out at 12:18°C with the temperature increasing to 15:20°C after 1 week. Humidity was uncontrolled. Seedlings were thinned to four evenly spaced plants per pot when the first true leaves were 10–12 mm long.

#### 2.5.2 Ryegrass

Low genetic variation *Lolium temulentum* (IBERS, Aberystwyth University seed stocks) were germinated on sand and seedlings pricked out into 5 × 8 grids in standard seed trays of John Innes compost containing 5% char by volume after 6 days when the coleoptiles were 2–3 cm long. Three replicates were established in August 2018 arranged as three random blocks across the staging in a glasshouse with natural lighting and temperature. The trays were subjected to free‐draining with manual watering to maintain moist compost at all times. Trays were rerandomized within the blocks weekly. Following top growth cut back, 6.5 g complete Vitax Q4 granular fertilizer (LBS Horticulture) was applied evenly per tray.

### 2.6 Plant trial growth measurements

#### 2.6.1 Lettuce

Germination (defined as the first appearance of the radicle/hypocotyl) was scored daily and G_50_ (the number of days for germination of eight or more seeds) was derived. The total number of seeds germinating was recorded and G_MAX_ (the number of days to maximum germination) also derived. The number of seedlings showing initial growth of the first true leaf and the number of seedlings with true leaves growing well when thinned were also recorded. At 21 days after sowing the largest seedling (only if true leaves were present) was removed from the pot. The state of the cotyledons was scored as 0 (dead/absent), 1 (dying/chlorotic and wilting), 2 (poor/yellowing), 3 (good/green and healthy). The number of true leaves was noted and the plant separated into root and shoot at the base of the hypocotyl. Total shoot fresh weight was measured. The roots were washed free of growing medium, blotted dry and fresh weight was recorded. Shoot and root were dried at 70°C to constant weight and reweighed. Dry matter (DM) content was calculated.

#### 2.6.2 Ryegrass

Measurements and growth scores were initially carried out at weekly intervals. Initial parameters assessed were as follows: the number of plants and tillers on the plants; the number of plants with subsequent leaves present; mean total leaf length (blade plus sheath from ‘ground’ level) of leaf 2 and leaf 3 on designated plants (*n* = 10 per tray). Due to differences in the growth rate and the presence of tillers, leaf measurements above were subsequently replaced by lamina length and width (mid‐leaf) of the youngest fully expanded leaf (ligule present) on the main or largest tiller for the designated plants (*n* = 10 per tray).

On day 35, plants were cut to a height of 4 cm with the biomass from each tray weighed, dried to constant weight at 70°C and reweighed. The number of plants showing regrowth on day 42 was scored and the length of regrowth on the tallest tiller of the designated plants measured (*n* = 10 per tray). Plant survival and regrowth after 2 weeks were scored again on day 49. The number of plants with at least one tiller regrowing and the number of these plants where the regrowth length was greater than 2 cm were counted. These counts were repeated for plants with three or more tillers. On day 77 the number of surviving plants was counted. Tiller number, reproductive development, lamina length and width (mid‐leaf) of the youngest fully expanded leaf (ligule present, and not excluding the flag leaf) on the main or largest tiller were recorded (*n* = 10 per tray). Reproductive development was scored as 0, plant dead; 1, vegetative; 2, stem elongating; 3, head just emerged; 4, head mid‐way emergence; 5, head fully emerged; 6, anthesis. Top growth for all plants was cut back to the base of the tillers at soil level and total biomass weighed, dried to constant weight at 70°C and reweighed.

### 2.7 Statistical analysis

Data were initially manipulated using Excel (Microsoft), then analysed in IBM SPSS v 25 (IBM Corp) using a Multivariate General Linear Model to produce MANOVAs with post hoc Tukey HSD multiple comparisons (*p* = .05).

## 3 RESULTS

### 3.1 Silage pH

Statistical analyses conducted on unspoilt silage materials (all containers minus one of the May mussel rope silos) demonstrated that there was a significant (*p* = .008) interaction between collection month and collection site in the mean end pH values of the silages (Table 2). There were significantly higher pH values for the May beach‐harvested macroalgae (predominantly *L. hyperborea* and *L. digitata*), the August cultivation line *L. digitata* and the August mussel line collection (predominantly *S. latissima*). All other treatments had a lower pH, with statistically the lowest pH occurring for July beach‐harvest collection (Table 1). The starting pH prior to ensilage was approximately 6.8 for all samples. All pH values declined during ensilage and the biological significance of these differences in slightly lower and slightly higher silage pHs was unclear.

**TABLE 2:** Mean pH values of ensiled macroalgae (*n* = 15 samples per Paxton container) produced from different collection months and locations

Collection month	Collection site			P values		
	Beach	Cultivation line	Mussel line	Collection month	Collection site	Collection month × Collection site
May	4.56^bc^	*	4.21^ab^	0.002	0.021	0.008
July	4.03^a^	4.14^ab^	4.22^ab^			
August	4.16^ab^	4.78^c^	4.56^bc^			

Note

Different lower case letters denote significant differences between pH values within each collection site using Tukey (*p* = .05).

* Missing value as insufficient macroalgae growth for ensiling for this location and date.

### 3.2 Compositional analysis of chars

Harvested kelp material was ensiled, dried, pelleted and underwent TCR pyrolysis with and without steam as detailed in the materials and methods. Proximate and ultimate analyses were conducted on the ensiled algae, regular char and steamed char, with the results shown in Table 3. These show the ensiled samples have very different values to those of the regular and steamed chars, which were more comparable to each other. As all values given were single, statistical analysis to determine differences was limited. However, despite the difference in their production, the regular char and steamed char values could be combined to statistically indicate the main differences between sources. The mean char values calculated are included at the bottom of Table 3. Significant differences detected between material sources are denoted in columns by lower case letters adjoining the mean char values. Generally, the results in Table 3 follow expected trends. Ensiled macroalgae pellets have a much higher proportion of H than the chars, they also have higher C and N proportions, moisture and HHV contents. Conversely, the silages have a much lower ash content than the chars. When looking at the mean char values, there is very little difference between the samples for each measured parameter. There were no significant differences between H, N, S, moisture or ash contents, across the analysed samples and with all O values at <1% there would be no significant difference here either. The carbon samples harvested in August (#6 to #8) are slightly higher than those harvested earlier in the year, but not significantly so. Higher values for HHVs and LHVs were seen in the August samples, but again not with a clear significant difference. Conversely, the average bulk density decreased in August samples compared to those collected earlier in the year, but differences were insufficient to provide a clear difference between August and the earlier months.

**TABLE 3:** Proximate and ultimate composition of silage and charred macroalgal pellets

Identifier	Elemental composition (% w/w of dry pellets)							MJ/kg		g/L
	C	H	N	S	O^†^	Moisture	Ash	HHV	LHV	Bulk density
S#1	32.2	3.9	1.1	1.4	35.9	13.8	25.6	13.6	12.7	669
S#2	36.0	4.0	1.7	0.9	28.1	10.3	29.4	12.0	11.2	710
S#3	34.7	3.9	1.2	0.7	35.3	13.7	24.3	13.2	12.3	643
S#4	32.9	4.4	1.4	1.4	34.1	10.9	25.8	13.1	12.1	686
S#5	33.3	4.3	2.1	1.2	33.3	10.9	28.9	13.1	12.2	700
S#6	40.2	5.2	1.2	0.8	32.2	10.3	20.5	15.8	14.6	632
S#7	36.3	1.1	1.1	1.1	39.4	12.5	21.1	14.2	13.9	649
S#8	33.4	4.5	1.7	1.1	36.3	14.3	23.0	13.6	12.6	646
RC#1	27.6	0.12	0.8	3.0	*	<0.1	71.8	9.8	9.8	354.6
RC#2	21.9	0.06	1.0	2.4	*	<0.1	73.5	7.7	7.7	490.8
RC#3	26.4	0.15	0.7	1.3	*	<0.1	69.8	9.0	9	314.0
RC#4	27.9	0.11	0.9	3.0	*	<0.1	71.1	9.7	9.7	473.1
RC#5	22.9	0.06	1.1	2.2	*	<0.1	76.9	8.4	8.4	431.0
RC#6	37.2	0.06	1.0	2.3	*	<0.1	69.5	9.6	9.5	327.4
RC#7	32.5	0.08	1.1	3.0	*	<0.1	65.9	11.9	11.9	260.6
RC#8	30.3	0.07	1.0	2.6	*	<0.1	71.6	11.2	11.2	260.6
SC #1	24.9	0.10	0.6	0.9	*	<0.1	76.2	8.4	8.3	324.8
SC #2	23.5	0.09	0.7	2.0	*	<0.1	75.2	7.2	7.2	417.6
SC #3	25.4	0.08	0.7	1.2	*	<0.1	74.1	7.6	7.5	281.6
SC #4	26.0	0.05	0.7	2.2	*	<0.1	77.1	8.6	8.6	455.8
SC #5	18.9	0.06	0.7	2.3	*	<0.1	80.7	8.0	8	419.0
SC #6	35.6	0.09	0.7	0.7	*	<0.1	77.7	9.6	9.5	307.2
SC #7	32.0	0.10	0.9	1.0	*	<0.1	68.9	11.6	11.6	263.0
SC #8	30.0	0.06	1.0	2.6	*	<0.1	73.8	10.4	10.4	258.6
$\bar{x}$ Ch #1	26.3abc	0.11a	0.7a	2.0a	*	*	74.0a	9.1ab	9.1ab	339.7ab
$\bar{x}$ Ch #2	22.7ab	0.08a	0.9a	2.2a	*	*	74.4a	7.5a	7.5a	454.2c
$\bar{x}$ Ch #3	25.9abc	0.12a	0.7a	1.3a	*	*	72.0a	8.3a	8.3ab	297.8a
$\bar{x}$ Ch #4	27.0bcd	0.08a	0.8a	2.6a	*	*	74.1a	9.2ab	9.2ab	464.5c
$\bar{x}$ Ch #5	20.9a	0.06a	0.9a	2.3a	*	*	78.8a	8.2a	8.2a	425.0bc
$\bar{x}$ Ch #6	36.4e	0.08a	0.9a	1.5a	*	*	73.6a	9.6abc	9.5abc	317.3a
$\bar{x}$ Ch #7	32.3de	0.09a	1.0a	2.0a	*	*	67.4a	11.8c	11.8c	261.8a
$\bar{x}$ Ch #8	30.2cd	0.07a	1.0a	2.6a	*	*	72.7a	10.8bc	10.8bc	259.6a

Note

Different lower case letters for the mean values in columns denote significant differences between samples using Tukey HSD at the 0.05 level. Commercial char SoilFixer density calculated as 359.5 g/L.

Abbreviations: C, carbon; H, hydrogen; HHV, higher heating value; LHV, lower heating value; N, nitrogen; O^†^, oxygen calculated by difference; RC, regular char; S, silage; S, sulphur; SC, steamed char; $\bar{x}$ Ch, mean of RC and SC.

* Not calculated due to skewing by high ash content.

Elemental analyses were also determined for the silage samples and the chars. These are shown in Table 4, with mean char values again determined and used for statistical analysis. These values are recorded in the lowest section of Table 4 with significant differences indicated in columns between the char sources denoted by lower case letters. In addition, the commercially available char used as a control in later experiments (SoilFixer) was also included for comparison. The main differences seen in Table 4 are between the silages and the macroalgae chars and between the commercial char and the macroalgae chars. As detailed in Table 3, there was a higher ash content in the chars than in the silage pellets, corresponding to a lower elemental content for all elements for the silage compared to the chars. The SoilFixer char, produced from coppice wood in the United Kingdom, had very different levels of some elements in it compared with the macroalgae. SoilFixer had approximately ×10 the Ba and Pb content and ×30 the Mn of the macroalgae chars. In contrast, the commercial char had ×10 less Ni and Sr, ×20 less K, ×50 less Cu and ×235 less Na. When examining the mean macroalgae char values only, the concentrations are generally much less varied. One set of mean char values that stands out here is #1, the May beach‐harvested material, with significantly higher concentrations of elements Al, Ba, Na and Zn but with significantly lower concentrations of Ca and Cd compared to other harvests. Interestingly, some chars varied considerably between regular char and steamed char for a sample, meaning that the increased elemental content for some harvests was not identified statistically. This included harvest #3 (July beach‐harvest) for Cr, Ni and Mo, where high levels of these elements were seen in the steamed char but not in the regular char. Conversely, for #1 (May beach‐harvest) high concentrations of Cd and Pb were seen in the regular char but not the steamed char. These differences are likely to be due to the relatively heterogeneous composition of the beach material and the fact that some of it was washed up onto the beach and therefore likely to be more heavily predated upon compared with the other sources of macroalgae.

**TABLE 4:** Mineral content of silages and chars determined by inductively coupled plasma spectrometer with atomic emission spectrometry (ICP‐AES) except for P, determined by absorbance at 740 nm

	Al	Ba	Ca	Cd	Cr	Cu	Fe	K	Mg	Mn	Mo	Na	Ni	P	Pb	Sr	Zn
	ppm	ppm	%	ppm	ppm	ppm	ppm	%	%	ppm	ppm	%	ppm	%	ppm	ppm	ppm
S#1	1,551.4	7.24	1.51	0.36	0.67	305.25	333.0	6.18	0.56	5.14	0.09	2.55	0.36	0.17	0.51	679.1	60.65
S#2	702.5	8.35	1.92	0.26	1.41	268.75	461.0	5.46	0.59	7.99	0.14	2.98	1.30	0.23	2.14	609.0	48.67
S#3	345.1	7.04	1.6	0.21	0.43	250.76	45.5	3.74	0.53	2.99	0.00	2.35	0.66	0.07	0.65	633.2	169.69
S#4	363.7	10.51	2.67	0.28	1.30	139.15	397.4	5.15	0.56	6.95	0.08	2.59	1.30	0.17	1.20	629.6	142.37
S#5	1,649.5	15.64	1.54	0.31	1.34	145.46	576.0	5.41	0.65	15.49	0.26	3.22	2.17	0.10	0.68	808.1	215.92
S#6	435.2	8.65	1.87	0.25	0.55	568.60	133.6	3.21	0.54	5.28	0.00	2.38	0.39	0.12	0.29	616.6	44.54
S#7	1,152.3	10.76	3.39	0.43	1.47	253.18	583.7	5.22	0.60	9.43	0.64	2.93	1.33	0.27	0.58	704.2	58.53
S#8	1,253.2	17.23	2.98	0.45	1.85	278.20	709.6	8.00	0.72	11.87	0.69	5.05	1.13	0.23	1.19	947.9	88.15
RC#1	5,061.5	61.61	3.49	1.13	16.05	395.97	1,725.9	13.89	1.26	52.32	1.6	9.83	8.35	0.21	14.44	1,809.5	662.36
RC#2	2,314.6	37.01	4.89	0.59	8.82	649.21	1,401.2	13.98	1.14	58.13	1.08	8.61	4.07	0.14	4.17	1,666.2	192.67
RC#3	2,388.8	38.29	4.94	0.14	3.92	598.98	809.9	14.26	1.10	15.91	0.28	7.87	2.05	0.31	1.46	1,453.3	258.78
RC#4	2,906.6	45.07	4.43	0.26	10.39	679.48	1,662.9	14.36	1.24	40.03	0.56	9.09	6.15	0.15	2.70	1,448.2	321.25
RC#5	2,538.4	41.63	7.43	0.48	6.87	551.3	1,565.4	13.62	1.12	38.82	0.97	8.11	3.41	0.28	2.79	1,466.4	181.31
RC#6	1,803.5	36.73	4.08	0.39	11.69	606.73	1,413.1	11.15	0.98	101.72	1.2	6.61	11.43	0.18	2.21	1,239.0	309.72
RC#7	1,138.5	26.67	4.14	0.25	4.63	728.59	585.0	12.47	1.12	12.81	0.27	8.28	1.93	0.19	1.42	1,596.6	341.21
RC#8	3,533.6	23.22	3.65	0.40	6.03	787.25	871.9	16.53	1.15	16.46	0.41	8.10	2.77	0.21	1.94	1,609.1	200.35
SC#1	4,148.6	54.44	3.99	0.40	8.68	357.23	1,686.1	14.10	1.33	57.62	1.08	9.62	4.54	0.11	1.59	1,634	602.72
SC#2	2,240.3	35.47	4.97	0.28	12.89	675.67	1,488.6	14.16	1.18	58.18	1.36	8.64	23.02	0.20	1.32	1,678.9	190.56
SC#3	2,692.3	42.11	5.21	0.28	280.97	642.26	3,207.0	15.49	1.21	94.58	3.59	8.29	1,018.21	0.23	0.93	1,574.4	336.69
SC#4	2,856.5	43.75	4.51	0.28	14.32	640.12	1,737.5	14.69	1.19	43.11	1.17	9.21	30.60	0.20	1.93	1,485.5	291.49
SC#5	2,843.8	43.92	7.92	0.25	8.09	784.05	1,412.7	14.57	1.23	33.41	1.78	8.76	25.12	0.19	1.93	1,597.1	277.44
SC#6	2,495.4	39.27	4.67	0.25	23.93	745.53	2,480.1	13.13	1.23	147.57	2.91	7.69	265.83	0.29	1.86	1,527.5	320.02
SC#7	1,229.5	42.95	4.27	0.08	3.58	757.54	596.9	12.94	1.17	14.19	0.72	8.61	1.44	0.25	0.86	1,636.5	352.58
SC#8	3,507.0	22.25	3.60	0.10	3.54	785.86	583.6	16.46	1.17	14.34	0.91	7.98	2.21	0.22	1.13	1,627.5	173.17
SoilFixer	2,600.7	386.35	4.60	1.16	6.51	13.61	6,565.9	0.68	0.41	1,453.41	0.33	0.04	8.39	0.15	28.34	169.7	108.72
$\bar{x}$ Ch #1	4,605.1d	58.0c	3.7a	0.8	12.4	376.6a	1,706.0	14.0abc	1.3	55.0ab	1.3	9.7c	6.5	1.6	8.0	1,721.8	632.5c
$\bar{x}$ Ch #2	2,277.5ab	36.2ab	4.9b	0.4	10.9	662.4b	1,444.9	14.1abc	1.2	58.2ab	1.2	8.6bc	13.6	1.7	2.8	1,672.6	191.6a
$\bar{x}$ Ch #3	2,540.6bc	40.2abc	5.1b	0.2	142.5	620.6ab	2,008.5	14.9bc	1.2	55.3ab	1.9	8.1ab	510.1	2.7	1.2	1,513.9	297.7ab
$\bar{x}$ Ch #4	2,881.6bc	44.4bc	4.5ab	0.3	12.4	659.8b	1,700.2	14.5abc	1.2	41.6ab	0.9	9.2bc	18.4	1.8	2.3	1,466.9	306.4ab
$\bar{x}$ Ch #5	2,691.1bc	42.8bc	7.7c	0.4	7.5	667.7b	1,489.1	14.1abc	1.2	36.1ab	1.4	8.4abc	14.3	2.3	2.4	1,531.8	229.4ab
$\bar{x}$ Ch #6	2,149.5ab	38.0ab	4.4ab	0.3	17.8	676.1b	1,946.6	12.1a	1.1	124.7b	2.1	7.2a	138.6	2.4	2.0	1,383.3	314.9ab
$\bar{x}$ Ch #7	1,184.0a	34.8ab	4.2ab	0.2	4.1	743.1b	591.0	12.7ab	1.2	13.5a	0.5	8.5abc	1.7	2.2	1.1	1,616.6	346.9b
$\bar{x}$ Ch #8	3,520.3cd	22.7a	3.6a	0.3	4.8	786.6b	727.8	16.5c	1.2	15.4a	0.7	8.0ab	2.5	2.1	1.5	1,618.3	186.8a

Abbreviations: Al, aluminium; Ba, barium; Ca, calcium; Cd, cadmium; Cr, chromium; Cu, copper; Fe, iron; K, potassium; Mg, magnesium; Mn, manganese; Na, sodium; Ni, nickel; P, phosphorus; Pb, lead; RC, regular char; S, silage; SC, steamed char; Sr, strontium; $\bar{x}$ Ch, mean of RC and SC; Zn, zinc.

Not calculated. For $\bar{x}$ Ch, lower case letters denote significant differences in columns at the *p* = .05 level by Tukey's multiple comparison test. Where no letters next to numbers = no significant difference occurs between mean values for that element.

### 3.3 pH and electrical conductivity

The chars were thoroughly immersed and shaken in deionized water before being centrifuged and the residual water tested for pH and EC. As before, the full results are shown in Table 5 with the mean of the two chars given at the bottom of the table and statistically analysed for differences between treatments.

**TABLE 5:** pH and electrical conductivity of charred macroalgal pellets

Treatment	pH	Conductivity (mS/cm)
RC#1	10.6	104.9
RC#2	10.7	107.3
RC#3	10.4	101.7
RC#4	10.6	106.0
RC#5	10.4	99.07
RC#6	9.9	73.5
RC#7	10.0	77.6
RC#8	10.0	96.2
SC#1	10.4	107.4
SC#2	10.4	96.3
SC#3	10.3	96.6
SC#4	10.4	103.1
SC#5	10.4	102.2
SC#6	10.0	88.7
SC#7	10.2	82.0
SC#8	10.0	101.4
SoilFixer	9.4	0.94
$\bar{x}$ Ch #1	10.5	106.2^bc^
$\bar{x}$ Ch #2	10.5	101.8^bc^
$\bar{x}$ Ch #3	10.4	99.1^b^
$\bar{x}$ Ch #4	10.5	104.6^bc^
$\bar{x}$ Ch #5	10.4	100.7^b^
$\bar{x}$ Ch #6	10.0	81.1^a^
$\bar{x}$ Ch #7	10.1	79.8^a^
$\bar{x}$ Ch #8	10.0	98.8^b^

Note

Different lower case letters for the mean values in columns denote significant differences between samples using Tukey HSD at the 0.05 level.

Abbreviations: RC, regular char; SC, steamed char; $\bar{x}$ Ch, mean of RC and SC.

There was no significant effect on pyrolysis method on pH and EC across the different chars (analysis not shown), therefore analyses values from char samples were combined for statistical analyses. There were no significant two‐way interactions for char pH, so no lower case signifiers are present for these values on Table 5. However, when compared by variable, there were significantly (*p* = .022) greater pH values noted for mussel line compared to beach and algae collection sites and significantly (*p* < .001) greater pH for May and July collection months compared to August. Biochar EC values demonstrated a significant (*p* < .001) interaction, with chars produced earlier in the season irrespective of the collection site and had a higher EC compared to char produced from seaweed collected in August (Table 5).

### 3.4 Plant trials

#### 3.4.1 Lettuce germination and growth

The initial trial used the different chars with a commercially available char as detailed in Table 1. Each was planted out with and without fertilizer. Table 6 details the overall char and fertilizer effects on the germination and early seedling development. Appendix Table A1 provides specific char values for germination scores with Appendix Table A2 showing final harvest values.

**TABLE 6:** Growth traits assessed in the germination and early growth of lettuce seeds, with and without the addition of macroalgae silage char and inorganic fertilizer

Growth trait	Without fertilizer		With fertilizer		Significance (*p*)		
	No char	With char	No char	With char	Char	Fertilizer	Char × Fertilizer
Germination							
G_50_	4.1 a	12.8 b	5.5 a	18.8 c	<.001	<.001	.001
G_MAX_	5.6 a	9.7 b	6.8 a	12.9 c	<.001	<.001	.029
Total germination	14.8 a	9.5 b	14.0 a	4.2 c	<.001	<.001	<.001
Plants growing leaf 1	14.0 a	1.1 b	13.0 a	0.9 b	<.001	NS	NS
Plants growing well	13.4 a	0.2 c	11.8 b	0.4 c	<.001	.026	.008
Final harvest (Day 21)							
Condition of cotyledons	2.8 a	0.6 b	3.0 a	0.3 b	<.001	NS	.016
Number of true leaves	4.9 a	0.5 b	5.5 c	0.4 b	<.001	.012	<.001
Shoot fresh weight (g)	1.12 a	0.02 b	1.75 c	0.02 b	<.001	<.001	<.001
Shoot dry weight (g)	0.086 a	0.002 b	0.118 c	0.002 b	<.001	<.001	<.001
Root fresh weight (g)	0.280 a	0.002 b	0.400 c	0.002 b	<.001	<.001	<.001
Root dry weight (g)	0.019 a	0.000 b	0.022 c	0.000 b	<.001	.001	.001
Shoot %DM	7.83	NA	6.69	NA	—	.003	—
Root %DM	6.81	NA	5.74	NA	—	.002	—
Shoot root ratio	4.88	NA	5.45	NA	—	NS	—

Note

Initial *n* = 16 for each treatment. G_50_ = the number of days taken for 50% of the seeds to germinate, G_MAX_ = days for maximum germination, cotyledon condition score range 0–3 where 0 = dead or absent, 1 = dying/chlorotic and wilting, 2 = poor/yellowing and 3 = good/green and healthy. Significance levels for main effect and interaction means from two‐way ANOVA (*n* = 54) are shown. Means followed by the same letter horizontally are not significantly different at the *p* = .05 level by Tukey's multiple comparison test. Derived traits (shoot and root dry matter content and shoot/root ratio) were only analysed by one‐way ANOVA for the effect of fertilizer.

Abbreviations: DM, dry matter; NA, not available; NS, not significant.

The macroalgae chars had a very clear adverse effect on lettuce germination and seedling growth. The addition of char to the growing medium increased the time it took for seeds to germinate, reduced total germination, reduced the number of seedlings which developed to produce the first true leaf and reduced the number of plants growing well when any thinning took place (Table 6. Further growth was severely restricted in the presence of char and only small weak seedlings, if any, were still alive 21 days after sowing. Across all the different chars tested there were significant effects of char, fertilizer and their interaction for all measured traits with the exception of the number of plants with initial growth of the first true leaf (where there was no char × fertilizer interaction) and the condition of the cotyledons at final harvest on day 21 (where there was an effect of fertilizer only; Table 6). The derived traits (shoot and root DM content and shoot: root ratio) could not be analysed by two‐way ANOVA because of the large number of missing values resulting from the low number of measurements for seedlings in the treatments including char; thus, only one‐way ANOVA for the effect of fertilizer is shown (Table 6).

The presence of any differences between the effects of the various char samples included in the study was examined using only data for seedlings grown with char and without fertilizer. There were no significant differences for the rate or extent of germination although all the macroalgae chars produced worse results than the commercial wood char control which gave data similar to the treatments without char (Appendix Table A1).

There were no significant differences between the chars on the rates of early growth compared to the SoilFixer control (Appendix Table A1) and none of the chars gave distinct significant differences when comparing growth traits at final harvest (Appendix Table A2). August‐harvested macroalgae regular and steam chars generally gave higher scores and weights than for chars from other months but again not to a significant level. Grouping the chars according to source of material (beach‐harvest, mussel line collection, algal line collection), harvest date (May, July, August) or process (regular char, steamed char) and analysing by one‐way ANOVA, did not show any significant effects for any of the measured germination or growth traits.

The underlying cause of the significant differences between the individual char treatments detected by ANOVA was, at least in part, the better performance of the seedlings grown with the SoilFixer control particularly during the early stages of the experiment (Appendix Table A1). The SoilFixer mean became less like the minus char controls as the experiment progressed (Appendix Tables A1 and A2). However, this may not be a real effect, but a confounding effect resulting from the experimental design (determined by the constraints of the growth cabinet facilities available) with all the pots containing the different chars standing on the same tray allowing liquid to be shared between all pots.

#### 3.4.2 Growth of ryegrass mini‐swards

Survival of ryegrass plants following transplanting was good, with virtually no seedling deaths in the first week (Table 7). Over the course of the growth trial very few plants died in the no addition control or the SoilFixer treatments (Table 7) though plants began to die early within the experiment in the macroalgae silage char treatments. Despite this, there were no significant differences between treatments with ANOVA until day 35. Even then the Tukey multiple comparison test did not show any pairs of treatments to be significantly different. By the final harvest on day 77 there were large and significant differences between treatments, with very good survival in the no addition control and SoilFixer treatments but very poor survival in treatments with the TCR chars, particularly those produced from silage made with mussel line sourced macroalgae (predominantly *S. latissima*). Leaf development was always slower in the macroalgae char treatments, again particularly with the chars produced from silage made with mussel line sourced *S. latissima*. Leaves 2, 3 and 4 all appeared earlier in the no addition control and SoilFixer treatments (Table 7). Additionally, the leaves in the no addition control and SoilFixer treatments were significantly longer than in the macroalgae char treatments except for leaf 1 on day 7 for the char produced from silage made with beach‐collected macroalgae (predominantly *L. digitata* and *L. hyperborea*). In fact, the plants grown with the char produced from silage made with beach‐collected macroalgae also showed greater survival and wider leaves than most of the other char treatments. Tiller development was always faster and greater in the no addition control and SoilFixer treatments than in all the different macroalgae char treatments (Table 7). Tiller numbers less than one result from the number of dead plants present in the mini‐sward. Regrowth after top growth was cut back and was also greater in the no addition control and SoilFixer treatments (Table 7). On day 42, the length of leaf regrowth was significantly greater in the no addition control and SoilFixer treatments than in all the macroalgae‐derived char treatments. On day 49 there were no pairs of treatments with significantly different live plants present, but there were significant differences in the number of plants showing substantial regrowth. Plants grown with the char produced from silage made with beach‐collected macroalgae again performed better than the other chars; the number of plants with one good growing tiller (length of leaf regrowth >2 cm) was not significantly lower than the no addition control. The no addition control and SoilFixer treatments yielded considerably more biomass although there were no significant effects on biomass DM content (Table 7). Fresh and DM production were both significantly greater than for all the macroalgae‐derived char treatments. Most plants were still in a vegetative state at the end of the experiment on day 77, although stem elongation was starting to occur in the no addition control and SoilFixer treatments. Reproductive scores less than one in the macroalgae silage char treatments result from the number of dead plants present in the mini‐sward.

**TABLE 7:** The effect of macroalgae silage pyrolysis chars on the growth of ryegrass mini‐swards

Trait		Controls		Regular char material			Steamed char material			Probability	
		No addition	SoilFixer	Beach‐harvest	Mussel line collection	Algal line collection	Beach‐harvest	Mussel line collection	Algal line collection	*p*	LSD 5%
Survival traits											
Number of live plants	Day 7	40.0	40.0	39.7	40.0	40.0	40.0	40.0	40.0	NS	
	Day 14	40.0	40.0	39.0	39.0	38.7	39.0	39.5	39.7	NS	
	Day 21	40.0	40.0	39.3	38.8	38.0	38.7	37.8	39.0	NS	
	Day 28	40.0	40.0	38.7	34.2	37.3	36.7	35.7	37.3	NS	
	Day 35	40.0 a	40.0 a	38.7 a	29.7 a	33.7 a	31.3 a	31.7 a	34.3 a	.043	7.26
	Day 49	40.0 a	40.0 a	38.0 a	27.5 a	32.7 a	29.7 a	26.0 a	32.3 a	.018	8.5
	Day 77	39.0 c	39.3 c	24.3 bc	10.1 ab	12.7 ab	19.0 ab	7.6 a	14.3 ab	<.001	9.66
Leaf traits											
Number of plants											
With leaf 2	Day 7	39.3 b	39.0 b	24.0 ab	8.2 a	15.3 a	11.3 a	10.2 a	17.3 ab	<.001	13.4
With leaf 2	Day 14	40.0 a	40.0 a	36.7 a	27.8 a	28.3 a	25.3 a	29.8 a	30.7 a	.035	9.79
With leaf 2	Day 21	40.0 a	40.0 a	38.0 a	30.7 a	33.3 a	30.7 a	32.7 a	32.7 a	.029	6.56
With leaf 3	Day 14	39.0 c	38.7 c	15.7 b	2.8 ab	5.7 ab	3.3 ab	2.3 a	6.7 ab	<.001	7.91
With leaf 3	Day 21	40.0 b	40.0 b	35.7 ab	23.4 a	28.0 ab	25.3 ab	23.9 ab	30.0 ab	.010	9.93
With leaf 4	Day 21	39.7 b	40.0 b	30.7 ab	5.1 a	16.7 ab	7.3 a	5.1 a	18.3 ab	<.001	15.50
Leaf length (mm)											
Of leaf 1	Day 7	148.0 b	154.2 b	110.2 ab	73.7 a	82.0 a	84.4 a	74.1 a	82.9 a	<.001	29.81
Of leaf 2	Day 14	251.5 b	253.6 b	117.7 a	19.6 a	59.0 a	44.1 a	33.2 a	66.3 a	<.001	64.03
Of leaf 2	Day 21	257.5 b	257.4 b	125.8 a	27.6 a	68.8 a	56.0 a	43.4 a	80.8 a	<.001	64.90
Of leaf 3	Day 21	316.4 b	312.8 b	131.4 a	23.3 a	64.6 a	43.5 a	16.7 a	82.2 a	<.001	80.64
Of the youngest fully expanded leaf	Day 28	289.1 b	287.0 b	134.5 a	23.2 a	69.2 a	50.0 a	32.7 a	87.2 a	<.001	71.31
	Day 35	342.1 b	335.1 b	170.9 a	35.2 a	96.7 a	70.4 a	33.9 a	109.9 a	<.001	92.18
	Day 77	264.2 b	258.9 b	64.5 a	7.0 a	43.0 a	78.8 a	4.2 a	43.1 a	<.001	50.42
Leaf width (mm)											
Of the youngest fully expanded leaf	Day 28	6.1 c	6.2 c	3.5 bc	0.6 a	1.9 ab	1.5 ab	1.1 ab	2.2 ab	<.001	1.62
	Day 35	6.8 b	6.9 b	4.0 ab	1.0 a	2.4 a	2.0 a	1.0 a	2.7 a	<.001	2.00
	Day 77	5.9 b	5.9 b	2.0 a	0.2 a	1.2 a	2.1 a	0.1 a	1.3 a	<.001	1.32
Tiller traits											
Number of plants with tillers	Day 21	39.0 b	39.7 b	15.3 a	0 a	6.3 a	2.7 a	2.4 a	8.3 a	<.001	11.08
Tiller number	Day 28	4.6 b	4.8 b	1.6 a	0.7 a	0.9 a	0.9 a	0.7 a	1.3 a	<.001	0.87
	Day 35	4.8 b	4.6 b	1.7 a	0.5 a	0.9 a	0.7 a	0.5 a	1.3 a	<.001	0.97
	Day 77	2.9 b	3.2 b	0.6 a	0 a	0.5 a	0.8 a	0 a	0.6 a	<.001	0.57
Regrowth traits											
Number of plants											
Regrowing after 7 days	Day 42	40.0 c	40.0 c	37.3 bc	25.5 a	25.7 a	28.0 ab	25.0 a	30.7 abc	<.001	7.01
With 1 tiller regrowing after 14 days	Day 49	40.0 b	40.0 b	35.0 ab	25.9 ab	27.0 ab	29.7 ab	19.4 a	26.7 ab	.008	10.2
With 1 tiller regrowth >2 cm	Day 49	40.0 b	40.0 b	27.0 ab	11.7 a	14.3 a	22.3 ab	12.7 a	16.3 a	<.001	11.2
With 3 or more tillers regrowing	Day 49	40.0 b	38.7 b	6.7 a	0 a	0.3 a	3.0 a	0.3 a	1.3 a	<.001	5.6
With 3 or more tillers regrowth >2 cm	Day 49	27.3 b	27.0 b	0 a	0 a	0.3 a	0.7 a	0 a	0.7 a	<.001	11.5
Length of leaf regrowth (mm)	Day 42	77.2 b	90.0 b	24.9 a	5.7 a	15.6 a	23.0 a	4.5 a	13.7 a	<.001	18.56
Reproductive traits											
Flowering score (0–6)	Day 77	1.7 bc	1.9 c	0.6 a	0 a	0.4 a	0.9 ab	0 a	0.5 a	<.001	0.57
Herbage production traits (g)											
Top‐growth fresh weight	Day 35	143.1 c	140.2 c	48.9 b	6.0 a	22.3 ab	15.7 ab	7.7 a	30.1 ab	<.001	22.36
Top‐growth dry weight	Day 35	12.2 c	12.1 c	4.7 b	0.7 a	2.3 ab	1.8 ab	0.9 a	3.1 ab	<.001	2.03
Top‐growth %DM	Day 35	8.6	8.6	9.8	10.5	10.2	12.2	10.9	10.3	NS	
Top‐growth fresh weight	Day 77	104.4 b	107.0 b	25.2 a	7.3 a	8.0 a	21.9 a	2.6 a	16.6 a	<.001	19.71
Top‐growth dry weight	Day 77	18.56 b	18.88 b	4.4 a	1.0 a	1.4 a	3.6 a	0.3 a	2.8 a	<.001	3.12
Top‐growth %DM	Day 77	17.9	17.6	17.7	16.4	19.0	16.7	18.4	18.3	NS	
Total dry matter production		30.8 c	31.0 c	9.1 b	1.6 a	3.7 ab	5.4 ab	1.2 a	5.9 ab	<.001	4.49

Note

Initial *n* = 40 transplanted seedlings per treatment tray. Flowering score = 0–6 where 0 = plant dead; 1 = vegetative; 2 = stem elongating; 3 = head just emerged; 4 = head mid‐way emergence; 5 = head fully emerged; 6 = anthesis. Significance level and 5% LSD of means from one‐way ANOVA (*n* = 3) are shown. DM, dry matter; NS, not significant. Means followed by the same letter are not significantly different at the *p* = .05 level by Tukey's multiple comparison test.

## 4 DISCUSSION

### 4.1 Statistical analysis

Large quantities of macroalgae were initially collected, filling 2 × 225 L ensilage containers per date and location (except May cultivated lines). Due to resource and practical constraints, it was not possible to collect more for each sample point. Each container held approximately 50 kg macroalgae when full but both containers had to be combined to produce sufficient pellets for both TCR char productions. The impact of this is not least seen in subsequent analysis, where combining regular and steam char values was not an ideal arrangement. However, as differences were seen between char sources, this provided greater clarity regarding where these differences occurred. Subsequently, further combinations of July and August chars were required to provide enough material to conduct the ryegrass growth trial. Though this reduced the number of samples further, it did not detract from the identified outcome in this study.

### 4.2 Composition

The proximate and ultimate data generated from chars of the differently harvested macroalgae following ensiling show trends typical of macroalgae compared to terrestrial biomass with relatively high ash content and low HHV and LHV. Moisture content for the chars is low compared to reported moisture contents for other pyrolysed macroalgae chars for example, for *Cladophora glomerata* samples pyrolysed between 300°C and 450°C, the moisture content was between 1.5% and 1.9% in subsequent analysis of the chars (Michalak, Basladynska, Mokrzycki, & Rutkowski, 2019). In this experiment, this is due to the analysis being conducted after samples were stored under nitrogen, minimizing exposure to water droplets in the air. Looking at the data more closely, within the ensiled samples there are no clear trends through the harvest season (May–August) or per harvest location for the ultimate or proximate data. The mean silage pH values in triplicate down the depths of the silage containers also did not show any distinctly different pH values between any of the macroalgae collections, though variation was present. May beach‐harvest and August cultivation line and mussel line had pH values between 4.5 and 4.8; the remainder of the ensiled samples had a pH <4.3. Satisfactory ensiling of land‐based herbage is considered to have a pH ≤4.4 within 7 days (Black, 1955) and is based on the ensiling concept of decreasing bacterial activity through lactic acid production (Redden et al., 2017). After this date, the pH sometimes gradually increases (Herrmann et al., 2015) so the pH values after 90 days along with the general appearance and smell of the silage indicate that all ensiled macroalgae achieved this initial pH drop. These pH values taken after 90 days ensiling fit within values seen in other studies such as pH 4.6 (*L. digitata* after 90 days) and 3.7 (*S. latissima* at day 90; Herrmann et al., 2015), though are higher than in others, for example pH 3.2–3.4 seen for *L. digitata* (Redden et al., 2017). The August‐harvested samples, with a higher mean pH for two out of three collections, could therefore be predicted to have been the poorer silages, but this did not translate into the subsequent char findings where there were no significant differences between the char pH values.

All chars were alkali, with pH values ranging from 9.9 to 10.7, compared to the SoilFixer control of 9.4. This increase in the macroalgae pH compared to the wood control was attributed to a combination of higher pyrolysis temperatures for the macroalgae chars and their higher mineral content which ranged from 65.9% to 80.7% (w/w) ash. This is comparative to previously published data, for example Bird, Wurster, Silva, Bass, and de Nys (2011) demonstrated macroalgae chars had an ash content ranging from 32.1% to 73.5% and a pH range from 7.8 to 10.1, depending on the species of seaweed pyrolysed. The seaweed chars produced in this research also had a higher ash content and pH compared to other pyrolysis feedstocks, for example rice straw with 30.6% ash and pH 10.8 (Zhang, Zhang, Yuan, Li, & Han, 2020).

Similarly to pH, increasing pyrolysis temperature and soluble mineral content of the biochar will lead to increases in EC value (Zhang et al., 2020). The EC on the macroalgae biochar was higher (73.5–107.4 mS/cm, dilution ration 1:5 biochar:deionized water) than previously published data for macroalgae chars which ranged from 15.3–61.2 mS/cm from saline macroalgae to 2.8 mS/cm for freshwater macroalgae, at a dilution ratio of 1:10 (Bird et al., 2011). Compared with other terrestrial pyrolysis feedstocks, macroalgae char had far higher EC values (Bird et al., 2011; Li et al., 2013) than other demonstrated EC values for example, 2.6–7.7 mS/cm for rice straw (1:5 dilution) and 0.815–2.0 mS/cm for cotton gin biochar (Rehrah et al., 2014), with far lower ash contents. The higher EC values may have arisen in the current research through a concentration of the mineral ash within the solid silage, as it dewatered during the ensiling stage. In August samples, there was lower ash content in the macroalgae char and this was reflected with the significantly lower EC values for August compared to the other two collection months.

When combining regular char and steamed char values, significant differences were seen in composition although these did not identify any collection method which was distinctly different from any other. Harvests differed more by season than by location (and by inference, by macroalgae species type), with August harvests generally higher in C with greater HHV and lower densities indicating a higher porosity. Together this and previously discussed results suggest that the overall composition of the different macroalgae species becomes relatively homogenous following ensiling and the two‐step TCR process, with seasonality a greater driver of composition change than differences in kelp species. This appears to be the first article where macroalgae silage has subsequently been processed by pyrolysis to char, thereby preventing comparison with the published literature. Terrestrial biomass has been used to produce silage and subsequently char in many papers (e.g. Corton et al., 2016; Sanger et al., 2017) but very few papers conducted comparisons between ensiled and fresh material which was subsequently pyrolysed, and when this did occur, additional steps were also included. For example, Tao et al. (2019) pyrolysed fresh and ensiled maize straw at a range of temperatures but only after the ensiled maize had been consumed by cattle and excreted. This leads to the unsatisfactory conclusion that until more work is conducted comparing chars produced from ensiled and comparative un‐ensiled biomass, a full understanding of parameters including composition, microstructure and elemental retention by these different chars will not be reached. This has implications in macroalgae species selection if cultivating for TCR, where if this pyrolysing process became more widespread, the emphasis regarding energy production should be on producing the maximum biomass yield possible rather than on the composition differences between species or strain types grown as the differences in the chars were not significant in this study.

For the elemental quantification data, one harvest stands out. Harvest #1 (May 2015 beach) is distinct from the other harvests in the chars, with significantly high concentrations of Al, Ba, Na and Zn, and low concentrations of Ca and Cu are present compared to other harvests. This is partly explained by char variation, where chars from this location harvested in May (#1) and July (#3) had large differences between regular char and steamed char values for some elements not seen elsewhere. For example, for the May (#1) harvest, significant increases of Pb ×9 and Cd ×3 were seen with the regular char giving higher values for heavy metals over the steamed char. For the July (#3) chars, this variation was reversed with the steamed char for Cr ×70 higher than for regular char; for Ni it was ×500 higher. Under similar tropical conditions, Chlorophyta (green) macroalgae took up a higher concentration of heavy metals than Phaeophyta (brown) macroalgae, which in turn absorbed the metals to a higher content than Rhodophyta (red) macroalgae (Al‐Shwafi & Rushdi, 2008). However in a separate study across the three ‘colours’ of macroalgae on lead only, brown seaweeds had the highest adsorption levels and red macroalgae the lowest across a pH range (Senthilkumar, Vijayaraghavan, Thilakavathi, Iyer, & Velan, 2007). Species selection clearly has a major role in uptake in these studies, but it is logical to presume that the wide variation in metal contents within the beach‐harvested material in this study was due to a greater range of seaweed species present from this collection site. In the other collection locations, the macroalgae was more homogeneous due to the environmental conditions around the growth and cultivation sites.

### 4.3 Plant trials

When the analysed individual elements are examined, all elements quantified within these samples are within the limit values for heavy metal concentrations in sludge for agricultural uses, the standard equating nearest to the addition of char to soil (EEC, 1986). However, the other aspect of elemental analysis is that of cumulative quantity. The total percentage mineral content of the SoilFixer char was 6.9%; for the macroalgae chars the average was 29.2%. This large proportion of metals is proposed to have contributed to the negative effect of the chars on the biomass grown with each of them, even if individually each metal concentration is not at toxic levels. When designing the experiment, after consideration, it was felt that having comparable quantities of char enabled a better comparison of the results to existing literature than if the quantity was altered to balance one or more of the elements, or the mean elemental content in the macroalgae char to that in the SoilFixer char. If the latter had occurred, a significantly smaller proportion of macroalgae char would have been added to the soil. This would potentially have confounded results by removing some of the additional positive effects seen when using char in soils such as the increase in microbial diversity and abundance in the soil (Nguyen et al., 2018).

The char in these studies was thus applied at a 5% (v/v) loading, which based on the average densities of John Innes compost no. 2 (J.I.M.A., 2010), grit sand (Walker, 2016) and perlite (Perlite.Info, accessed 2019) results in 20.3 g char per kg soil or 2% (w/w). It is a comparable addition to a number of other studies such as in Abujabhah, Bound, Doyle, and Bowman (2016) where it equalled the lower test proportion of 2% w/w char on soil microbial communities and the lower test quantity in a 1 and 10 kg/m^2^ germination trial addition (Solaiman, Murphy, & Abbott, 2012); other studies have equated using 1 kg/m^2^ char to 2% (w/w) or 1.5% (w/w) depending on the soil used (Van Zwieten et al., 2010). It is almost half of that used in one study (Bird et al., 2012), where 3.5% (w/w) char was added to the soil. There is a good summary table in a review by Agegnehu, Srivastava, and Bird (2017) showing application rates within which values used in this study are again comparable or below those used in other studies.

For both the lettuce germination and ryegrass mini‐sward growth trials, inclusion of the macroalgae chars had a universally detrimental effect. With the lettuce, negative impacts ranged across all parameters assessed, from a longer germination time leading to poorer germination, to fewer true leaves appearing and a lower average plant health score. At the final harvest, those with char had fewer leaves, were in a worse state of health and were consequently significantly lower yielding. Interestingly, the inclusion of fertilizer with the char had a further negative effect on the seedlings, delaying germination and reducing total seedling number, reducing true leaf number though it did marginally increase seedling health scores. This indicates that one problematic aspect of char inclusion as proposed above could have been too high a total elemental level present, so addition of more through the fertilizer exacerbated the issue for the seeds and seedlings. This ‘toxic’ effect of high inorganics has been previously seen (Andresen, Peiter, & Kupper, 2018) with other toxic aspects identified as pH and salinity (Mumme et al., 2018). The pH of the macroalgae chars was up to 1.5 pH units higher than the SoilFixer commercial char, as discussed in the composition section above, but salinity has not yet been considered. When calculating the elemental proportions seen in Table 4 into mmol addition within the soil, the highest addition is that of sodium (Na), at 36.8 mmol (mean char addition). The effect of salt stress is therefore a possibility, despite enzymes within plants being typically only inhibited by NaCl at higher concentrations of approximately 100 mmol/L (Ketehouli et al., 2019), as different species have variable levels of salt tolerance. For example, Na^+^ was shown to significantly lengthen sunflower germination time and reduce germination velocity from concentrations of 25 mmol or more (Wu, Jiao, & Shui, 2015), which is lower than the concentrations found in this study. The actions of salt stress on plants are many but include a reduction of CO_2_ uptake through the closure of stomata and an imbalance of ions within the cytosol, with an excess of Na^+^ and Cl^−^ arising whilst becoming deficient in K^+^ (Bose et al., 2017). This causes an increase of reactive oxygen species or antioxidants from the chloroplasts and mitochondria, in turn causing the formation of antioxidants (Ketehouli et al., 2019). The second highest element in this study was K^+^, with soil concentrations of a comparable value of 36.1 mmol, so inhibitions for this element are less likely to be problematic here than in other environments but overavailability may also create issues.

The problems surrounding the interpretation of this data are the wide range of elements present at relatively high concentrations within the char and subsequently within the soil. Though there have been many papers and reviews looking at individual elements and especially the effect of salinity on a wide range of crops (Chen et al., 2018; Machado & Serralheiro, 2017; Ren et al., 2020; Wu et al., 2015), relatively few papers combine the effect of salt stress with the presence of other elements on plant growth. Chen et al. (2018) claims to be the first paper studying the effect of NaCl and Mn together on cotton growth. Here they found that there was an antagonistic effect when both compounds were present on growth and yield, meaning that the plants showed inhibition of growth or yield at higher concentrations of combined NaCl and Mn than that occurred with the compounds individually. They concluded by postulating that trace elements in saline water could alleviate some of the negative impacts of salt stress on the plant. In the work reported in this paper, there are a wide range of trace elements present which could reduce some of the effects of the Na^+^ and other high‐concentration elements but to what extent and how much the cumulative content of these elements have an overall negative effect is difficult to determine without a multielemental pot trial to compare them at different concentrations to one another. Finally, it is important to consider that the inclusion of these elements do not just affect the plants in the study but also the microcosm surrounding them including the fungi and soil bacteria (Venancio et al., 2017) which will in turn affect the soil quality.

Using residual char produced from a pyrolysis process as an agricultural fertilizer is a viable concept, but in our studies we have shown it to generate universally negative impacts on plant germination and growth. The chars were applied at comparable volume and weight proportions to those used in multiple other studies, but created a poor environment for the plants to grow in. The authors propose that two additional factors are involved in the comparison and interpretation of this data. One is that of the feedstock source; macroalgae have high ash contents with different elemental proportions compared with terrestrial biomass. A 2% w/w macroalgae char has a different elemental balance provision for germinating and growing plants compared with char produced from terrestrial materials such as wood, straw or sawdust, as seen by the cumulative average elemental values given for the macroalgae char being more than ×4 higher than the SoilFixer wood‐derived commercial char used in this study. High proportions of elements such as sodium retained in the macroalgae char from the marine environment may also have a negative effect through increasing soil salinity.

The other factor is that the quality of the char is not conducive to either germination or plant growth. A number of researchers have identified that lower pyrolysis temperatures generate char which is beneficial to plants, whereas chars produced using higher pyrolysis conditions decreased germination (Roberts & de Nys, 2016; Roberts et al., 2015). This is supported by the control SoilFixer char for the lettuce seedlings which gave good initial germination and overall grew much better than seedlings germinating in TCR‐char soils, but which showed a decrease in health and growth of the seedlings as the experiment progressed. The shared water tray was proposed as the reason for this change, with components from the macroalgae char leaching into the tray and affecting the control plants. This was confirmed when the control was included for the rye grass trial in a separate tray; on this occasion the negative impact was not seen. This suggests that one effect of the macroalgae‐derived chars may be the leaching of these elements into nearby agricultural lands and water systems, to the detriment of plants beyond the trial area.

The wider‐ranging impact of this study is to highlight that as the bioeconomy grows, so too do the process‐generated residues. The subsequent use or disposal of biological residues following initial processing is too often left as a hypothetical solution by academics and in business plans. The danger is that these assumptions may be wrong, as this study shows, where it is demonstrated that in addition to positive‐growth controls, some chars have very negative impacts on plant germination and/or growth.

## APPENDIX 1

**TABLE A1:** The effect of the different char samples on germination and initial seedling growth of lettuce without additional fertilizer

Char sample	G_50_ (days)	G_MAX_ (days)	Total germination (number)	Plants growing leaf 1 (number)	Plants growing well (number)
Mean without char	4.1	5.6	14.8	14.0	13.4
RC#1	12.3	10.3	9.0	0.0 a	0.0 a
RC#2	12.7	9.7	10.3	0.3 a	0.0 a
RC#3	12.3	9.0	11.7	0.3 a	0.0 a
RC#4	17.0	9.3	6.3	0.0 a	0.0 a
RC#5	13.3	9.7	7.0	0.0 a	0.0 a
RC#6	7.7	9.7	13.3	2.7 a	0.0 a
RC#7	8.0	9.3	11.0	1.3 a	0.0 a
RC#8	17.0	10.3	8.0	1.0 a	0.0 a
SC #1	17.0	9.7	7.0	0.0 a	0.0 a
SC #2	17.0	11.0	9.0	0.0 a	0.0 a
SC #3	12.7	9.0	10.3	1.0 a	0.0 a
SC #4	14.0	10	8.0	0.0 a	0.0 a
SC #5	17.3	9.3	6.0	0.0 a	0.0 a
SC #6	11.3	10.7	11.3	1.0 a	0.0 a
SC #7	12.0	10.3	10.3	2.0 a	0.0 a
SC #8	8.3	9.3	10.3	1.3 a	0.0 a
SoilFixer	4.3	6.3	14.7	11.7b	5.5 b
*p*	NS	NS	NS	<.001	.048

Note

Initial seed *n* = 16. G_50_ = the time taken for 50% of the seeds to germinate, G_MAX_ = the time for maximum germination. Significance levels for main effect means from one‐way ANOVA (*n* = 3) are shown. Means followed by the same letter vertically are not significantly different at the *p* = .05 level by Tukey's multiple comparison test.

Abbreviations: NS, not statistically significant; RC, regular char; SC, steamed char.

**TABLE A2:** Effect of the different char samples on growth traits measured at final harvest after 21 days without additional fertilizer

Char sample	Condition of cotyledons (score)	Number of true leaves	Shoot fresh weight (g)	Shoot dry weight (g)	Root fresh weight (g)	Root dry weight (g)
Without char mean	2.8	4.9	1.12	0.086	0.28	0.019
RC#1	0.0	0.0 a	0.00 a	0.000 a	0.00 a	0.000 a
RC#2	0.7	0.3 ab	0.01 a	0.001 a	0.00 a	0.000 a
RC#3	0.7	0.3 ab	0.01 a	0.001 a	0.00 a	0.000 a
RC#4	0.0	0.0 a	0.00 a	0.000 a	0.00 a	0.000 a
RC#5	0.0	0.0 a	0.00 a	0.000 a	0.00 a	0.000 a
RC#6	2.0	1.7 ab	0.05 a	0.005 ab	0.01 a	0.001 ab
RC#7	1.3	1.0 ab	0.04 a	0.004 ab	0.00 a	0.000 a
RC#8	0.7	0.3 ab	0.02 a	0.002 a	0.00 a	0.000 a
SC #1	0.0	0.0 a	0.00 a	0.000 a	0.00 a	0.000 a
SC #2	0.0	0.0 a	0.00 a	0.000 a	0.00 a	0.000 a
SC #3	0.7	0.7 ab	0.02 a	0.002 ab	0.00 a	0.000 a
SC #4	0.0	0.0 a	0.00 a	0.000 a	0.00 a	0.000 a
SC #5	0.0	0.0 a	0.00 a	0.000 a	0.00 a	0.000 a
SC #6	0.7	0.0 a	0.00 a	0.000 a	0.00 a	0.000 a
SC #7	1.3	1.0 ab	0.05 a	0.004 ab	0.00 a	0.001 ab
SC #8	1.3	1.3 ab	0.03 a	0.003 ab	0.00 a	0.000 a
SoilFixer	1.0	2.0 b	0.05 a	0.008 b	0.02 b	0.001 b
*p*	NS	.002	.039	.005	<.001	.001

Note

Initial seed number = 16, cotyledon condition scored 0–3 where 0 = dead or absent, 1 = dying/chlorotic and wilting, 2 = poor/yellowing and 3 = good/green and healthy. Significance levels for main effect means from one‐way ANOVA (*n* = 3) are shown. Means followed by the same letter vertically are not significantly different at the *p* = .05 level by Tukey's multiple comparison test.

Abbreviations: NS, not statistically significant; RC, regular char; SC, steamed char.
